# Prevalence of alcohol consumption and related factors among students of higher education centers in one of the northeastern cities of Iran

**DOI:** 10.3934/publichealth.2019.4.523

**Published:** 2019-12-02

**Authors:** Behrad Pourmohammadi, Moahammad Ali Jalilvand

**Affiliations:** 1 Research Center for Health Sciences and Technologies, Semnan University of Medical Sciences, Semnan, Iran; 2 Student Research Committee, School of Management and Medical Information Sciences, Isfahan University of Medical Sciences, Isfahan, Iran

**Keywords:** alcohol drinking, students, personal satisfaction, Iran

## Abstract

**Introduction:**

Knowing the prevalence and the factors associated with alcohol abuse among students can be an important step in initiating preventive measures. The purpose of this study was to determine the prevalence of alcohol consumption and its related factors in students of higher education centers in one of the northeastern cities of Iran in 2017.

**Methods:**

This descriptive-analytical study was performed on 668 students from 7 higher education centers. The samples were selected by stratified random sampling. The valid researcher-made questionnaires were issued to the subjects, and were collected immediately after being filled out. The obtained data were analyzed using appropriate statistical tests.

**Results:**

Ninety-two (13.77%) students had history of alcohol consumption, with 55.6% continued to drink alcohol. The mean age of the consumers was 23.92 years, of whom 77.41% were male, 75% were single, 55.43% were non-indigenous, and 74.46% had history of smoking. 81.52% of the subjects were undergraduates, while 36.95% studied mathematics and engineering. There was a significant relationship between alcohol consumption and age, gender, GPA, being non-indigenous, personal residence, smoking, history of alcohol consumption in family and friends, satisfaction with academic major and city in which they study (p < 0.05).

**Conclusion:**

The findings showed that alcohol consumption was relatively high in students of higher education centers and many variables (ten out of fifteen studied variables) were involved in this process. Therefore, careful planning and serious measures are needed to prevent this problem.

## 1. Introduction

In 2016, mortality resulting from alcohol consumption was higher than that caused by diseases such as tuberculosis, HIV/AIDS and diabetes. 3 million deaths (5.3% of all deaths) worldwide and 132.6 million disability-adjusted life years (DALYs)—i.e. 5.1% of all DALYs in that year caused by alcohol consuming [1]. Studies on the economic outcomes of alcohol consumption have shown that the costs of lost productivity and the associated criminal costs accounted for more than 1% of countries' GDP [2]. The consumption of alcohol and the complexity of its side effects have made the monitoring of rate and pattern of alcohol consumption as a key part of health policy [3].

Alcoholic beverages are the most common substances in all age groups over 15, and their misuse is classified as harmful to health. In a 2013 ranking of the top 25 leading health risk factors in the world, alcohol was in the 6^th^ place [4]. In various countries, alcohol consumption has been recognized as one of the students' behaviors [5]. University students report exciting and invigorating experiences throughout their university lives. These experiences are linked with stressful times due to academic work load, competition among peers and pressure to succeed [6].

Drinking alcohol increases the risk of wide range of social harms to students. There are numerous reports of links between alcohol and tobacco consumption, absenteeism, academic failure, extending the education and still being incomplete, high-risk sexual behaviors, and violence among students [7]–[10]. Alcohol consumption may also continue in the post-school years and may show its consequences as dependency, poor performance at work, and inability to find a job [5].

There are several factors associated with alcohol consumption among students including demographic characteristics such as age and gender; socioeconomic status including income and parents' education level; lifestyle factors such as personal health, physical activity, nutritional information and perceived quality of life, social activities and study related stress; factors related to cultural and ethnic differences; alcohol consumption by parents; family relationships; peer pressure; stress in the educational environment; and even genetics [9],[11]–[15].

Although alcohol prevalence is generally lower in Muslim countries than in non-Muslim countries, its contribution to the national burden of health cannot be ignored [5]. Due to the prohibition of alcohol consumption in Islam, information on alcohol consumption among students is limited, and the pattern of alcohol consumption in these countries differs greatly from the pattern in non-Islamic countries [6].

In various studies, the rate of alcohol consumption among Iranian adolescents and students has been reported 2–68%. These rates showed a consumption level higher than the normal level of society, and they also proved that the consumption level could be variable in different locations and groups [3]. Knowing the prevalence and the factors associated with alcohol abuse among students can be an important step in initiating preventive measures. Therefore, regarding the above-mentioned issues and the importance of this issue, the present study aimed to determine the status of alcohol consumption and its related factors among students of higher education centers in one of the north-eastern cities of Iran in 2017.

## 2. Materials and methods

It was a cross-sectional descriptive-analytical study which was conducted in 2017. The study population consisted of 668 students from seven higher education centers. The samples were selected through stratified random sampling. After obtaining the necessary permissions, the list of students in each higher education center was obtained and then according to the number of students, a proportion of the total sample size was assigned to each center. After defining a number for each student, students were selected from each center using simple random sampling per calculated quota. If a student did not wish to participate in the study or was not available at the sampling time, a substitution would be made by simple random sampling. Data collecting tool was a researcher-made questionnaire. In this study, considering the cultural conditions of Iran, any history of alcohol use in lifetime was defined as “alcohol consumption” In order to prepare the questionnaire, after reviewing the literature and consulting with experts in this field, a question bank was designed. Then after merging, omitting and editing the questions, a draft questionnaire was sent to 10 experts to determine the content validity. After collecting the experts' opinions, the questionnaire was distributed among 100 samples, and its reliability was confirmed by Cronbach's alpha test (Cronbach's alpha was 0.839 after omission of dissimilar questions). The questionnaire consisted of three sections and 36 questions which included 16 questions about demographic information, 12 questions about pattern of alcohol consumption, and 8 questions about the factors affecting alcohol consumption. The appearance of questionnaire was designed to reassure the subjects of confidentiality, in a way that information was not readable from the cover. Data were collected by a trained questioner. Ethical considerations, in addition to the written form, were re-informed to the participants. The data were analyzed using descriptive statistics (centrality and dispersion indices) and analytical statistics (Kolmogorov-Smirnov test, independent-samples and paired-samples T-test, ANOVA, Chi-square test) at the significant level of P < 0.05. In this regard STATA software, version 13, was used. Due to the prohibition of alcohol consumption in Iran, any alcohol drinking in this study is considered as abuse.

## 3. Results

The mean age of subjects was 24.78 years. 49.05% of them were male and the rest were female. Their mean grade point average (GPA) was 15.78 (out of 20) at the time of the survey and the average household size was 5.12. 6.46% of students were associate students, 77.76% were undergraduate, 14.03% were getting their masters and 1.73% were PhD students. In terms of marital status, 69.88% were single, 28.36% were married, and 1.75% were in other status (divorced and widowed). 54.45% of the students were indigenous and 45.54% were non-indigenous (Table 1).

44.36% of the subjects lived in their homes with their parents; 28.12% lived in dormitories; 10.67% lived lonely on their own; and 16.84% lived in other conditions. In terms of father's education, 48.48% of the fathers were illiterate; 18.56% had elementary education; 18.40% had secondary school education, 31.20% had high school diploma, and 27.36% had higher education. In terms of mother's education, 49.7% of the mothers were illiterate; 23.61% had elementary education; 17.42% had secondary school education; 34.36% had high school diploma; and 17.10% had higher education. 19.63% of students had history of tobacco consumption, while 13.77% reported that there was an alcoholic in their family, and 27.20% reported that one of their friends was an alcoholic. 55.37% of students stated their awareness of alcohol was high and very high. The satisfaction with the city in which they were studying was very high in 38.38% of students, in 34.75% was high, in 23.85% was moderate, and it was low and very low in the rest of subjects. In terms of university satisfaction, 13.67% were very highly satisfied, 24.48% were highly, 38.19% were moderately, 12.87% were little and 10.17% were very little satisfied. Regarding their satisfaction with their academic major, 20.25% were very highly satisfied, 19.77% were highly satisfied, 30.53% were moderately satisfied, and the rest were little or very little satisfied (Table 2).

**Table 1.** Relationship between demographic variables and alcohol consumption history among students of studied higher education centers in 2017.

Variable		Alcohol consumption history		Total subjects (668 subjects)	P-value
		Yes (92 subjects)	No (576 subjects)		
Age	Mean ± S.D	23.92 ± 4.5	24.58 ± 6.5	24.48 ± 6.3	0.001
GPA from 20	Mean ± S.D	15.15 ± 2.3	15.88 ± 2.2	15.78 ± 1.85	0.025
Household size	Mean ± S.D	4.91 ± 1.5	5.13 ± 1.71	5.09 ± 1.7	0.433
Birth order	Mean ± S.D	2.24 ± 1.4	2.62 ± 1.70	2.57 ± 1.7	0.843
Gender	Male	72 (77.41)	265 (44.61)	342 (49.05)	0.001
	Female	21 (22.58)	329 (55.38)	350 (50.94)	
Education level	Associate	6 (6.652)	35 (6.45)	41 (6.46)	0.591
	Undergraduate	75 (81.52)	418 (77.12)	493 (77.76)	
	Postgraduate	11 (11.95)	78 (81.52)	89 (14.03)	
	PhD	0	11 (11.95)	11 (1.73)	
Academic major	Medical sciences	14 (15.21)	51 (8.70)	65 (9.58)	0.235
	Humanities	32 (34.95)	239 (40.78)	272 (39.97)	
	Mathematics & Engineering	34 (36.95)	221 (37.00)	255 (37.61)	
	other	12 (13.04)	75 (12.79)	87 (12.83)	
Marital status	Single	69 (75.00)	409 (69.08)	478 (69.88)	0.069
	Married	19 (20.65)	175 (29.56)	194 (28.36)	
	Other	4 (4.34)	8 (1.35)	12 (1.75)	
Residential place	Indigenous	41 (44.65)	326 (56.01)	367 (54.45)	0.027
	Non-indigenous	51 (55.43)	326 (43.98)	307 (45.54)	

92 students (13.77%) had a history of alcohol consumption and 55.6% of them still continued to drink alcohol. The mean age of starting alcohol consumption among students was 18.8 years. 77.41% of alcohol user students were male, 75% were single, 55.43% were non-indigenous, and 74.46% had history of smoking. 43.33% of alcohol consumers lived with their parents, and 55.55% had no personal income and their parents paid their education fees. 81.52% of them were undergraduates, and 36.95% of them were majoring in mathematics and engineering. 50% and 84.33% of alcohol consumer had history of alcohol consumption by family members and friends, respectively. 42.52% of consumer students reported moderate satisfaction with university; and 34.88% had very high level of satisfaction with the city; and 31.39% had very little satisfaction with their academic major (Tables 1 and 2).

**Table 2.** Relationship between social and behavioral variables and alcohol consumption history among students of studied higher education centers in 2017.

Variable		Alcohol consumption history		Total subjects (668 subjects)	P-value
		Yes (92 subjects)	No (576 subjects)		
Residence	With parents	39 (43/33)	256 (44/52)	295 (44/36)	0.000
	Dormitory	19 (21/11)	168 (29/21)	187 (28/12)	
	By his own	24 (26/66)	47 (8/17)	71 (10/67)	
	Other	8 (8/88)	104 (8/17)	112 (10/67)	
Income source	In person	35 (38/88)	137 (28/82)	172 (25/86)	0.009
	Parents	50 (55/55)	404 (38/88)	454 (68/27)	
	Other	5 (5/55)	34 (5/91)	39 (5/86)	
Father's education	Illiterate	6 (6/81)	22 (4/09)	28 (4/48)	0.143
	Primary school	11 (12/5)	105 (19/55)	116 (18/56)	
	Secondary school	28 (31/81)	103 (19/18)	115 (18/40)	
	High school	28 (31/81)	167 (31/09)	195 (31/20)	
	Higher education	31 (35/22)	140 (31/09)	171 (27/36)	
Mother's education	Illiterate	8 (9/30)	38 (7/19)	46 (7/49)	0.266
	Primary school	18 (20/93)	127 (24/05)	145 (23/61)	
	Secondary school	9 (10/46)	98 (18/56)	107 (17/42)	
	High school	32 (37/20)	179 (33/90)	211 (34/36)	
	Higher education	19 (22/09)	86 (16/28)	105 (17/10)	
Smoking history	Yes	70 (74/46)	70 (11/30)	140 (19/63)	0.000
	No	24 (25/53)	549 (83/75)	573 (80/36)	
Alcohol abuse in family	Yes	40 (50/00)	33 (7/33)	73 (13/77)	0.000
	No	33 (41/25)	351 (78/00)	384 (72/45)	
	Don't know	7 (8/75)	66 (14/66)	73 (13/77)	
Alcohol abuse by friends	Yes	70 (84/33)	81 (17/16)	151 (27/20)	0.000
	No	4 (4/83)	231 (48/94)	235 (42/34)	
	Don't know	9 (10/84)	160 (33/89)	169 (30/45)	
Knowledge about alcohol	Too much	42 (49/41)	137 (25/46)	179 (28/73)	0.000
	Much	23 (27/05)	143 (26/57)	166 (26/64)	
	Moderate	17 (20/00)	205 (38/10)	222 (35/63)	
	Little	1 (1/17)	38 (7/06)	39 (6/26)	
	Very little	2 (2/35)	15 (2/78)	17 (2/72)	
Satisfaction with the city	Very high	30 (34/88)	194 (35/46)	224 (35/38)	0.001
	High	23 (26/74)	197 (36/01)	220 (34/75)	
	Moderate	22 (25/58)	129 (23/58)	151 (23/85)	
	Little	2 (2/32)	16 (2/92)	18 (2/84)	
	Very little	9 (10/46)	11 (2/01)	20 (3/15)	
Satisfaction with the university	Very high	8 (9/19)	78 (14/39)	86 (13/67)	0.096
	High	17 (19/54)	137 (25/27)	1544 (24/48)	
	Moderate	37 (42/52)	207 (38/19)	244 (38/79)	
	Little	10 (11/45)	71 (13/09)	81 (12/87)	
	Very little	15 (17/24)	49 (9/04)	64 (10/17)	
Satisfaction with the academic major	Very high	11 (12/79)	117 (21/42)	128 (20/25)	0.001
	High	12 (13/95)	113 (20/69)	125 (19/77)	
	Moderate	25 (29/06)	168 (30/76)	193 (30/53)	
	Little	11 (12/79)	73 (13/36)	84 (13/29)	
	Very little	27 (31/39)	75 (13/73)	102 (16/13)	

There was a significant inverse association between alcohol consumption and age; in a way that alcohol consumption was lower in the older age groups (p = 0.001). Besides, alcohol consumption was more common in students with lower GPA (p = 0.025). The history of alcohol consumption was significantly higher in male (p = 0.001) and non-indigenous students (p = 0.027) (Table 1).

Alcohol consumption was significantly higher among subjects who lived in their own homes (p = 0.000). Alcohol consumption was higher among students whose parents paid their education fees (p = 0.009). Moreover, there was a significant direct relationship between smoking history and alcohol consumption (p = 0.000). Subjects whose friends or family members had history of alcohol abuse reported significantly more alcohol consumption (p = 0.000). Furthermore, alcohol consumption history was more seen in students who were more satisfied with the city or their academic major (p = 0.001) (Table 2).

There was no significant relationship between alcohol consumption and household size, birth order, educational level, academic major, marital status, parental education, and university satisfaction (Tables 1 and 2).

## 4. Discussion

The percentage of students in this study reporting a history of alcohol consumption was 13.77%. This was in line with the findings of Motevalian et al. (2015) study, which ranged 6.7–20% in different age groups of students at Tehran University of Medical Sciences [16]. However, this figure was more than the findings of some other Iranian researchers, such as Abbasi-Ghahramanloo et al. (2015), Rezahosseini et al. (2014), and Mardani et al. (2012), respectively, who reported the prevalence of alcohol abuse among medical students in Iran 6.9%, in Rafsanjan 5.7%, and in Bandar Abbas Azad University 9.9%. The figure was also more than the findings from other countries researchers, such as Al-Ameri et al. (2016) and Almousavi (2014), respectively from Baghdad and Karbala with 9.7% and 2%, and Ruisoto et al. (2016) from Ecuador with 8.7%. On the other hand, this figure was less than the findings of other researchers in other countries who investigated the history of alcohol abuse in students, including the studies of Pillai et al. (2014) from Nitte University in Karnataka, India with 26.4%, Al-Mousavi et al. (2009) from Iraq with nearly 30%, Seipone et al. (2013) from Molepolole University in Botswana with 58.4%, Cherian et al. (2014), Kyei and Ramagona (2013) from South Africa with 72.5% and 65%, respectively, and Zadarko et al. (2018) from the European Carpathian region countries (Poland, Slovenia, Ukraine and Romania) with 80% [17]–[27]. The findings of the present study indicated a high level of alcohol consumption among the students of higher education centers in the studied city compared to the students of universities in other parts of the country, despite the illegal and unauthorized consumption of alcohol in Iran. These differences could be due to various factors such as sample size, research method, mostly non-indigenous students, cultural and social conditions of the city, etc. Moreover, cultural, social, legal, and religious differences could justify the differences to other countries, including those that had a consumption level higher than Iran.

Investigating the relationship between gender and alcohol consumption in the present study showed that alcohol consumption was higher among male students. This finding was consistent with the findings of some Iranian researchers, including Motevalian et al. (2015), Mardani et al. (2012), Sohrabi et al. (2012), Abbasi-Ghahramanloo et al. (2015), Rezahosseini et al. (2014); and it was also consistent with the findings of researchers from other countries, including Wilsnack et al. (2009) from England, Schulte et al. (2005) from USA, Ruisoto et al. (2016) from Ecuador, Mphele et al. (2013) from Botswana, Rawa Jaafar and et al. (2016) from Iraq, and Ghandour et al. (2014) from Lebanon [7],[19],[21],[24],[28]–[31]. This has led some studies to consider the male gender as a risk factor for alcohol abuse. Religious factors and social behaviors could have been involved; nevertheless, there were some exceptions, for example, in a country like the United States, there was no gender difference in alcohol abuse among Muslims [21]. Perhaps it was due to the origin of individuals and adaptation of them to the community in which they live.

In the present study, the association between age and alcohol abuse among students was significant. This was in line with a study by Mohammadkhani (2012) [31]. But it was different from the findings of Sohrabi et al. (2012) study [28], although Sohrabi's study was conducted in the high school students group, which could justify the difference.

In this study, the age of drinking onset among students was 18.8 years, which was in line with the findings of Sohrabi et al. (2012), who reported the age of drinking onset at 18 years [28]; while it was not consistent with the results of Mohammadkhani's study, which reported the age of drinking onset at 14 years [31]. However, since the study was conducted among high school students, this difference could be attributed to the difference between the target groups. In the study of Al-Ameri et al. (2016) in Iraq, the highest alcohol consumption among students was reported in the age group of 20 to 24 years. Sanchez-Ramirez et al. (2017) also reported the hazardous age of drinking in Canada and Australia from 18 to 24 years old. This age range was reported by Bendtsen et al. (2011) in Sweden from 18 to 29 years [19],[32],[33]. However, it seems that there is a high level of alcohol consumption in the age of 18–29. Being non-indigenous could affect different aspects of students' physical and mental health due to being apart from family and lifestyle changes. In the present study, non-indigenous students reported more alcohol abuse. But Sohrabi et al. (2012) study on the public university students showed a different status [28]. However, it should be noted that more than half of the alcohol-consuming students in the present study (55.43%) were non-indigenous and lived apart from their families in various forms (dormitory, living lonely on their own, living with relatives, etc.). Students' lives away from their families could be a risk factor for alcohol abuse. This was also seen in the findings of Al-Mousavi et al. (2009) from Iraq, Ghandour et al. (2014) from Lebanon, Abbasi-Ghahramanloo et al. (2015), as well as Rezahosseini et al. (2014) both from Iran [17],[18],[20],[30].

Findings of the present study clearly showed that there was a reverse association between alcohol consumption and GPA. These findings were consistent with those of Ghaderi et al. (2014) and Ansari et al. (2007) [34],[35]. The relationship between high-risk behaviors and GPA could be bilateral. Both high-risk behaviors such as alcohol consumption or substance abuse could impair educational attainments, and both academic failure and lowered GPA could increase the risk of high-risk behaviors. Various studies have been conducted for many years worldwide about the multiple effects of tobacco consumption on health. Smoking can be a prelude to many health risk behaviors and illegal drug abuse. One thing to note in recent years is that these two substances (cigarettes and alcohol) can synergistically cause various diseases [36],[37]. In the present study, there was a direct relationship between alcohol consumption and smoking. These findings were similar to those of Ansari et al. (2007) and Mohammadkhani (2012) [31],[35].

History of substance or alcohol consumption among friends or family members could change the attitude and increase the desire and access to consume. Being accepted into peer groups has been a strong motivation for adolescents and young people to abuse alcohol or substance. This was confirmed by the findings of the present study. Findings of the present study, like the studies by Mohammadkhani (2012) and Mardani et al. (2012), showed that alcohol consumption by friends and family members could be a risk factor for students' alcohol abuse [27],[31].

In the present study, there was a significant relationship between students' satisfaction with their academic major, the city in which they studied and alcohol consumption. These findings were in contrast to the findings of the study by Murphy et al. (2005), conducted on students in the United States. No significant difference was found between alcohol consumption and academic satisfaction in male and female students; however, there was a significant association between social satisfaction and alcohol consumption in male students, while no significant association was found in female students [38]. In Murphy et al. (2005) study, Alfonso's and Pavot's quality of life questionnaires were used; whereas, in the present study, considering the numerous variables, questioning was performed in just two parts to examine student's satisfaction with city and academic major.

In the present study, there was no significant relationship between marital status and history of alcohol Consumption , which was not consistent with the findings of Mardani et al. (2012) study that showed a significant relationship between alcohol and substance abuse and being single [27]. Perhaps the difference in the number of higher education centers in both studies justified this difference, for example, Mardani's research was conducted in just one university, but the present study was conducted in 7 universities with more discipline diversity and different educational conditions.

Moreover, there was no significant difference between the education level and alcohol consumption in the present study. This was different from the findings of Ghaderi et al. study (2014) in Jiroft [34], in which postgraduate students reported more alcohol consumption. Differences in geographic characteristics and differences in accessibility could account for this difference in results.

One of the limitations of the present study was that some centers were not interested in cooperating in running the project, but with numerous follow-ups and explaining the importance of the issue and the confidentiality of the information, they were satisfied with the cooperation.

## 5. Conclusion

The research findings exhibited that alcohol consumption was relatively higher than expected in the students of higher education centers in comparison with the other similar studies among Iranian students, and also several variables including; being non-indigenous, student's income source, smoking history, history of alcohol abuse by family and friends, and dissatisfaction with the academic major were shown to be involved in this process. Providing necessary training on the risks of alcohol consumption, precision in choosing the academic major, and selecting indigenous students can be effective in preventing and reducing alcohol consumption.
